# Evaluating the Safety of Gastrointestinal Contrast Agents in Pediatric Patients: A Retrospective Study

**DOI:** 10.7759/cureus.97727

**Published:** 2025-11-25

**Authors:** Abdullah Alshehri, Rayan Hejles, Mohammad Aldaffaa, Raif Nassir, Wassima Kaidali, Yasser Alfraih, Tariq Altokhais

**Affiliations:** 1 Pediatric Surgery, King Saud University, Riyadh, SAU; 2 Pediatric Surgery, Ad Diriyah Hospital, Riyadh, SAU; 3 Pediatric Surgery, King Khalid University Hospital, Riyadh, SAU; 4 Pediatric Surgery, King Salman Medical City, Madinah, SAU; 5 College of Medicine, Alfaisal University, Riyadh, SAU

**Keywords:** barium, contrast medium, fluoroscopy, infants, necrotizing enterocolitis

## Abstract

Background: Gastrointestinal (GI) contrast imaging is commonly performed in pediatric patients to diagnose and treat various GI conditions. However, a widely held clinical belief suggests that contrast agents may have deleterious side effects on the intestinal wall, particularly in premature infants or those with pre-existing intestinal inflammation. This presumed risk is primarily supported by anecdotal reports and older animal studies.

Objective: This study aimed to investigate the potential association between GI contrast agent exposure and the development of necrotizing enterocolitis (NEC) within a 30-day period in infants.

Materials and methods: A retrospective study was conducted at a single tertiary care institution with a level IV neonatal intensive care unit. All 245 infants who underwent any form of GI contrast-enhanced radiological study were included. The primary outcome was the development of NEC within 30 days following exposure to a contrast agent.

Results: A total of 245 patients were included, of whom 15 (6.1%) had a documented history of NEC occurring more than two weeks prior to undergoing contrast imaging. Only one term newborn suspected of NEC progressed to intestinal perforation following contrast agent exposure.

Conclusion: Our findings suggest that GI contrast agent exposure is generally safe in infants and does not increase the risk of NEC development within 30 days, including premature newborns. However, it may be safer to avoid hyperosmolar contrast agents in patients with active NEC.

## Introduction

Necrotizing enterocolitis (NEC) is a syndrome of severe intestinal inflammation and necrosis that predominantly affects premature and low-birth-weight infants (LBWIs). Although the precise etiology of NEC remains unclear, prematurity is a major risk factor for its development. This susceptibility is most likely due to the immature nature of the intestinal tract in preterm infants and those with intrauterine growth restriction (IUGR), which is characterized by diminished peristalsis, elevated permeability, and reduced secretion of gastric acid and digestive enzymes. Thus, the intestinal mucosa in preterm infants is vulnerable to injury and deterioration under inflammatory conditions, culminating in extensive necrosis and perforation [1,2].

Neonatologists and pediatric surgeons believe that gastrointestinal (GI) contrast agents, administered either orally or rectally, may be associated with the development or progression of NEC, particularly in premature infants [3,4]. The mechanism of contrast-induced GI injury is thought to mirror that of feeding-related injury, with osmolarity playing a role. High-osmolar feeding formulas or fluids administered orally can prompt an intraluminal fluid shift, leading to mucosal injury and potentially the development of NEC [3,5].

An illustrative case from 1980 by Grantmyre et al. documented a neonate who underwent a hyperosmolar contrast enema for meconium evacuation due to meconium ileus and subsequently developed NEC with perforation attributed to contrast agent exposure [5]. Similarly, in 1986, Negrette et al. reported a case series involving 16 neonates with suspected NEC who underwent lower GI contrast enemas for diagnostic purposes. Two neonates experienced colonic perforation that was attributed to the contrast agent [6]. Despite these anecdotal observations, empirical evidence substantiating the causal link between GI contrast agent exposure and NEC development in children remains limited.

Thus, our study aims to investigate the potential association between GI contrast agent exposure and the development of NEC within 30 days of the contrast administration period in children under one year of age.

## Materials and methods

A retrospective cohort study was conducted after obtaining all necessary ethical approvals from the Institutional Review Board. The study was conducted in a tertiary care institution with a level IV neonatal intensive care unit (NICU).

All patients aged one year or younger who underwent at least one GI contrast radiological study between January 2015 and December 2020 were included. Patients who had contrast studies performed at other institutions were excluded due to unavailability of data.

Analyzed data included prenatal (maternal age, mode of delivery, IUGR) and postnatal variables (APGAR score, gestational age, age, sex, weight, history of NEC). Details of the contrast agent, including type, volume, and indication, were collected. The primary outcome was the occurrence of NEC within 30 d following exposure to a GI contrast agent. The diagnosis of NEC was based on the clinical assessment of the treating physician. Additionally, any unexplained feeding interruption within 30 days following the contrast study was investigated as potential marker of NEC. For inpatients, daily progress notes and assessments were reviewed; for outpatients, clinic and emergency department records were examined for visits following contrast imaging

## Results

This study included a cohort of 245 patients, of whom 145 (59.2%) were male patients. The mean gestational age was 35.6 ± 4 weeks (range 23.2-42.5 weeks), and the mean birth weight was 2,393 ± 883 g (range: 500-4,240 g). Table 1 summarizes the baseline characteristics of the cohort. Among the study population, 15 patients (6.1%) had a history of NEC that occurred more than two weeks before undergoing contrast imaging.

**Table 1:** Baseline characteristics of the study population (N = 245). IUGR, intrauterine growth restriction; GDM, gestational diabetes mellitus; C/S, caesarean section.

Variables	Mean ± SD (range) or frequency (%)
Prenatal data	
Maternal age (years)	31.9 + 6.4 (18–46)
Polyhydramnios	15 (6.1%)
Oligohydramnios	5 (2%)
Pre-eclampsia	3 (1.2%)
IUGR	25 (10.2%)
GDM	22 (8.9%)
Delivery by C/S	109 (44.5%)
Postnatal data	
Gestation age (weeks)	35.6 + 4.0 (23.2–42.5)
APGAR Score at 1 min	6.4 + 1.8 (1–8)
APGAR Score at 5 min	8.2 + 1.4 (2–10)
Birthweight (g)	2,393 + 883 (500–4,240)
Neonatal age at first feed (d)	3.3 + 7.1 (0–48)

Section A in Table 2 presents the baseline data of patients within two weeks prior to contrast imaging. Forty-two patients (17.1%) had feeding intolerance unrelated to NEC. The occurrence of infections or the requirement for mechanical ventilation, inotropic support, or antibiotics within two weeks before contrast imaging is also summarized. As shown in section B in Table 2, 192 patients (78.4%) were feeding normally within 24 hours before contrast administration.

**Table 2:** Clinical status of patients at two time intervals: (A) Within two weeks before the contrast study and (B) Within 24 h before the contrast study.

Variables	Frequency (%)
A. Clinical data within 2 weeks before the contrast study	
Feeding intolerance	42 (17.1%)
Mechanical ventilation	11 (4.5%)
Inotropes	1 (0.4%)
Antibiotics use	51 (20.8%)
Infection	5 (2%)
Thrombocytopenia	8 (3.3%)
B. Clinical data within 24 h before the contrast study	
Normal feeding	192 (78.4%)
Infection	20 (8.2%)
Antibiotics use	52 (21.2%)

Details of the contrast imaging are summarized in Table 3. A total of 222 patients (90%) had a weight above 2 kg, and 171 patients (70%) were older than two months of age of contrast imaging. A total of 245 contrast imaging studies were performed, of which 155 (63.3%) were upper gastrointestinal contrast studies, and 27 (11%) were small bowel follow-throughs. The most commonly used contrast agent was iohexol (Omnipaque™, GE HealthCare, Chicago, IL, U.S.A), used in 140 studies (57.1%), followed by diatrizoate (Gastrografin™, Bayer Healthcare Pharmaceuticals, Germany) and barium sulfate, each used in 13 studies (5.3%). The average volume of contrast agent used was 46.9 ± 27.5 mL (range, 3-170 mL). Gastroesophageal reflux disease (GERD) was the primary indication for contrast imaging in 118 patients (48.2%). Feeding was resumed 5.27 ± 5.8 d (range, 0-30 d) following the contrast imaging.

**Table 3:** Clinical data at the time of contrast imaging and procedure details.

Variables	Frequency (%) or mean ± SD (range)	
Weight (grams)		
0–1,500	19 (7.7%)	
1,501–2,000	5 (2.0%)	
2,001–2,500	100 (40.6%)	
> 2,501	122 (49.6%)	
Age (days)		
≥ 30	36 (14.7%)	
> 31 to ≤ 60	38 (15.5%)	
> 61 to ≤ 90	25 (10.2%)	
> 91	146 (59.6%)	
Contrast imaging type		
Modified barium swallow	15 (6.1%)	
Esophagogram	10 (4.1%)	
Upper GI study	155 (63.3%)	
Small bowel follow-through	27 (11%)	
Colostogram	21 (8.6%)	
Contrast Enema	17 (7.0%)	
Contrast agent		
Diatrizoate (Gastrografin™)	13 (5.3%)	
Lohexol (Omnipaque™)	140 (57.1%)	
Barium sulfate	13 (5.3%)	
Other/Unknown agent	79 (32.2%)	
Contrast volume (mL)	46.9 + 27.5 (3–170)	
Study indications		
Meconium disease	1 (0.4%)	
Hirschsprung disease	9 (3.7%)	
Anorectal malformation	6 (2.4%)	
Malrotation	7 (2.9%)	
Feeding intolerance	17 (6.9%)	
Gastroesophageal reflux disease (GERD)	118 (48.2%)	
Others/unknown	87 (35.5%)	

Within 30 days of administration of a GI contrast agent, one patient developed NEC five days after exposure. This patient was a term newborn to a healthy 32-year-old mother, with a birth weight of 2,650 g. After feeding was initiated, the baby exhibited signs of sepsis, with a possibility of Bell’s stage 1A NEC. On day 4 of life, the patient underwent a small bowel follow-through contrast study to investigate abdominal distension and feeding intolerance. The type and volume of contrast agent used were not documented. The imaging study did not reveal any clear gastrointestinal pathology, and feeding was resumed. However, by day 9 of life, the patient developed severe sepsis with pneumoperitoneum and underwent emergency surgery for perforated NEC.

## Discussion

The use of contrast agents in pediatric GI imaging is pivotal in diagnosing and managing various conditions. As shown in Table 4, several contrast agents have been developed with differing characteristics, including osmolality, water solubility, density, viscosity, barium versus iodine-based, and safety in cases of suspected intestinal perforation [7,8]. Historically, concerns have been raised regarding the use of GI contrast media in high-risk pediatric populations such as premature infants or those with suspected intestinal inflammation. Some contrast agents are thought to induce endoluminal fluid shifts and/or intestinal inflammation, which could be detrimental in specific cases [5,6,9].

Early studies provided insights into the potential risk of GI contrast agent exposure and the development of NEC. Lutzger et al. demonstrated significant colonic inflammation and necrosis in rats following Gastrografin enemas, attributing this effect to the Tween 80 component rather than the agent’s osmolality. The observed inflammation was not identified in specimens where other contrast agents, namely, Hypaque-25 or barium sulfate, were used [10]. Similarly, Feigenberg et al. observed severe mucosal injury and high mortality rates in neonatal rats exposed to high-osmolarity Gastrografin, less than a week after the start of the experiment [11]. Furthermore, McAlister et al. reported acute inflammatory responses on the peritoneal surface of rats injected with iodine-containing contrast media [12]. Conversely, Ratcliffe et al. noted no complications in 115 GI tract examinations using low-osmolality, water-soluble contrast agents such as ioxaglate (Hexabrix), iohexol (Omnipaque), and iopamidol (Niopam) in 89 children [13].

**Table 4:** Types of commonly used contrast media in gastrointestinal (GI) imaging.

Contrast agent type	Contrast agent name(s)	Osmolality (mOsm/kg)	Water solubility
Barium sulfate	E-Z-HD®, E-Z-Paque®, Liquid Polibar Plus®, Readi-cat-2®, Micropaque®	N/A (Suspension, not a solution)	Insoluble
Iodinated	Gastrografin	1,500–2,100 (High osmolality)	Water soluble
	Conray-30	~1,200–1,800	Water soluble
	Omnipaque (Iohexol)	~300–700 (Low osmolality)	Water soluble
	Visipaque (Iodixanol)	~290 (Iso-osmolar)	Water soluble

Several case reports have further fueled the debate on the potential association between GI contrast agent exposure and the development of NEC. Tuladhar et al. documented Gram-negative sepsis in a premature infant with a prior NEC diagnosis following oral administration of Gastrografin, suggesting mucosal injury induced by the contrast agent [9]. Conversely, Leonadis et al. found no evidence directly attributing NEC development to barium sulfate contrast enemas in term neonates presenting with GI symptoms. Despite the typically aggressive course of NEC in term infants, their patients demonstrated favorable outcomes following contrast studies, akin to the findings in our study [14].

Our findings align with previous studies, as almost all patients diagnosed with NEC prior to contrast agent exposure did not experience disease progression after exposure. The only exception was a single-term newborn who developed NEC following exposure to an unidentified contrast agent. However, the causal relationship between exposure to contrast agents and NEC progression remains inconclusive in this case.

A recent randomized controlled trial by Haiden et al. investigated Gastrografin use for meconium evacuation in premature and LBWIs, noting a higher, albeit statistically insignificant, NEC incidence in the intervention group. Nevertheless, infants in the intervention group exhibited earlier meconium evacuation and enteral feeding initiation, along with reduced hospital stays, underscoring the multifaceted considerations surrounding contrast agent use [3].

Hospital guidelines by the American Academy of Pediatrics in 1974 recommended that feeding osmolarity should not exceed 300-400 mOsmol, which is equivalent to that of breast milk, warning that higher concentrations could cause GI injury. This consensus suggested that high-osmolarity contrast agents cause mucosal breakdown, loss of intestinal barrier, and the development of NEC. These cautions have led to the adoption of iso-osmolar agents and contrast dilution protocols to mitigate such risks [3,4]. Despite ongoing debate regarding the exact mechanisms underlying NEC development in relation to contrast agent exposure, our findings emphasize the importance of careful clinical decision-making in neonates undergoing contrast studies.

Our study is limited by its retrospective design, relatively small sample size, and incomplete data on contrast agent, which might underpower the study for possibly a very rare outcome. Additionally, establishing causation in this design is not possible.

## Conclusions

While GI contrast studies are a cornerstone of pediatric GI diagnostics, their role in NEC development remains ambiguous. Our findings suggest that contrast exposure is generally safe in appropriately selected infants without possible risk factors. However, hyperosmolar contrast agents are best avoided in pediatric patients with suspected NEC until further well-designed studies establish their safety in this high-risk population.
